# SIRT3-Mediated CypD-K166 Deacetylation Alleviates Neuropathic Pain by Improving Mitochondrial Dysfunction and Inhibiting Oxidative Stress

**DOI:** 10.1155/2022/4722647

**Published:** 2022-09-01

**Authors:** Binbin Yan, Qiang Liu, Xiaobao Ding, Yuwen Lin, Xiaowei Jiao, Yuqing Wu, Huihui Miao, Chenghua Zhou

**Affiliations:** ^1^Jiangsu Key Laboratory of New Drug Research and Clinical Pharmacy, Xuzhou Medical University, Xuzhou 221004, China; ^2^Jiangsu Province Key Laboratory of Anesthesiology, NMPA Key Laboratory for Research and Evaluation of Narcotic and Psychotropic Drugs, Xuzhou Medical University, Xuzhou 221004, China; ^3^Department of Anesthesiology, Beijing Shijitan Hospital, Capital Medical University, Beijing 100038, China

## Abstract

Numerous studies have shown that mitochondrial dysfunction manifested by increased mitochondrial permeability transition pore (mPTP) opening and reactive oxygen species (ROS) level, and decreased mitochondrial membrane potential (MMP) plays an important role in the development of neuropathic pain. Sirtuin3 (SIRT3), a nicotinamide adenine dinucleotide (NAD^+^)-dependent histone deacetylase, has been shown to inhibit mitochondrial oxidative stress. However, the role of SIRT3 in neuropathic pain is unclear. In this study, we found that the protein and mRNA levels of SIRT3 were significantly downregulated in the spinal cords of spared nerve injury- (SNI-) induced neuropathic pain mice, while overexpression of spinal SIRT3 reversed SNI-induced pain hypersensitivity. Further study showed that SIRT3 overexpression reduced the acetylation level of lysine 166 (K166) on cyclophilin D (CypD), the regulatory component of the mPTP, inhibited the mPTP opening, decreased ROS and malondialdehyde (MDA) levels, and increased MMP and manganese superoxide dismutase (MnSOD) in SNI mice. Point mutation of K166 to arginine on CypD (CypD-K166R) abrogated SNI-induced mitochondrial dysfunction and neuropathic pain in mice. Moreover, inhibiting mPTP opening by cyclosporin A (CsA) improved mitochondrial function and neuropathic pain in SNI mice. Together, these data show that SIRT3 is necessary to prevent neuropathic pain by deacetylating CypD-K166 and further improving mitochondrial dysfunction. This study may shed light on a potential drug target for the treatment of neuropathic pain.

## 1. Introduction

Neuropathic pain (NP) is a kind of chronic pain caused directly by the injury or disease of somatosensory nervous system. It is manifested as spontaneous pain, hyperalgesia, abnormal pain, and paresthesia and other clinical characteristics [1]. It severely affects about 7%-8% of the European population and causes a significant medical burden worldwide [2]. However, the etiology of neuropathic pain is complex and its pathogenesis is still unclear.

Mitochondria are important intracellular organelles, which can provide energy for cellular activities through oxidative phosphorylation. It has been reported that mitochondria are not only the main target of reactive oxygen species (ROS) but also an important source of ROS [3]. In pathological states, increased ROS level causes oxidative stress, leading to the opening of the mitochondrial permeability transition pore (mPTP), a non-specific pore located between the inner and outer membrane of mitochondria. The increased opening of mPTP may lead to a decrease of mitochondrial membrane potential (MMP) and a burst of ROS production, forming a vicious cycle and further aggravating mitochondria dysfunction [3, 4]. A large number of studies have shown that mitochondrial dysfunction and oxidative stress are involved in the occurrence and development of neuropathic pain [4–6]. In addition, intraperitoneal injection of cyclosporin A (CsA), a specific inhibitor of mPTP, can improve this condition and alleviate allodynia and hyperalgesia in animals with neuropathic pain [7]. Furthermore, intrathecal injection of tert-butyl hydroperoxide (t-BOOH), a reactive oxygen donor, induced painful behavior in normal mice [8, 9]. In contrast, free radical scavengers and antioxidants were effective in alleviating pain behavior in animals with neuropathic pain [10]. These findings suggest that improving mitochondrial dysfunction and inhibiting oxidative stress are particularly promising potential drug targets for the treatment of neuropathic pain.

The mPTP is mainly composed of adenine nucleotide translocator (ANT) located in the inner membrane of mitochondria, voltage-dependent anion channel (VDAC) located in the outer membrane of mitochondria, and cyclophilin D (CypD) located in the matrix of mitochondria. It can regulate the permeability of mitochondrial membrane and maintain the relative stability of mitochondrial internal environment [11–13]. It is reported that CypD can inhibit the opening of mPTP after being bound, resulting in significant improvement in cardiac function after ischemia-reperfusion [14]. Therefore, the opening of mPTP may be the trigger of mitochondrial dysfunction and CypD is the key to the opening of mPTP.

In recent years, an increasing number of studies have shown that histone acetylation plays an important role in neuropathic pain [15, 16]. SIRT3 is a deacetylase dependent on nicotinamide adenine dinucleotide (NAD^+^), which is mainly distributed in tissues and organs in mitochondria. Recent studies have found that SIRT3 can regulate cellular stress response, metabolism, senescence, and apoptosis. For example, SIRT3 can influence mitochondrial oxidative stress response by regulating the acetylation levels of related proteins in mitochondria [17]. Overexpression of SIRT3 can reduce the acetylation level of CypD, thereby alleviating postoperative cognitive dysfunction in sepsis rats [18]. In addition, age-related cardiac hypertrophy can be inhibited by SIRT3-mediated deacetylation of CypD [19]. Moreover, the acetylation of lysine 166 (K166) on CypD has been demonstrated to be a key target of SIRT3 [19, 20]. However, whether SIRT3 is involved in the development of neuropathic pain by deacetylating CypD-K166 and subsequently improving mitochondrial dysfunction and inhibiting oxidative stress is unknown.

Therefore, in this study, we explored the role of SIRT3-mediated deacetylation of CypD-K166 in the occurrence of neuropathic pain in mice and its underlying mechanism.

## 2. Materials and Methods

### 2.1. Animals

Adult (6-8 weeks, weighing 20-30 g) male C57BL/6J mice were provided by the Animal Center of Xuzhou Medical University. B6/JNJU-PPIF^em1Cin^(K166R)/Gpt gene mice were provided by Gempharmatech Co., Ltd. The brief procedure is as follows: sgRNA was transcribed in vitro and the donor vector was constructed. Cas9, sgRNA, and donor were microinjected into the fertilized eggs of C57BL/6J mice for homologous recombination and F0 generation mice were obtained. The positive F0 generation mice were verified by PCR and sequencing generation mice were mated with C57BL/6J mice to obtain a positive F1 generation mouse model with stable inheritance. A total of 153 mice were used in this study and all animals were maintained 4~5 per cage under controlled conditions (23-25°C, 40%-50% relative humidity, 6 am to 6 pm alternative light/dark cycles) with food and water *ad libitum*. The animal care and laboratory procedures were approved by the Animal Care and Use Committee of Xuzhou Medical University (approval number: 202006A140).

### 2.2. SNI Mouse Model

**Figure 1:**
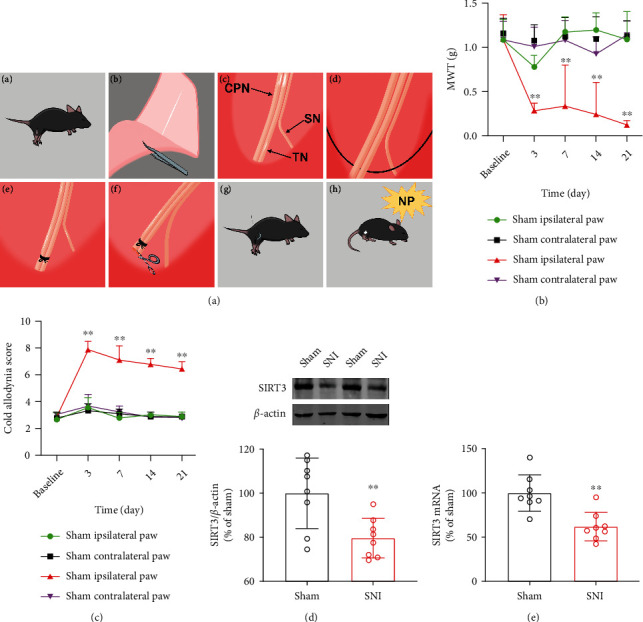
SIRT3 expression is downregulated in the spinal cords of mice with neuropathic pain. (a) SNI surgical procedures. (b) Mechanical withdrawal threshold (MWT) in ipsilateral paw and contralateral paw of SNI and Sham group mice before and 3, 7, 14, and 21 d after surgery (*n* =9. ^∗∗^*P* < 0.01 vs Sham ipsilateral paw). (c) Cold allodynia score (*n* =9. ^∗∗^*P* < 0.01 vs Sham ipsilateral paw). (d) Western blots for SIRT3 protein (*n* =8. ^∗∗^*P* < 0.01 vs Sham). (e) Real-time quantitative PCR for SIRT3 mRNA (*n* =8. ^∗∗^*P* < 0.01 vs Sham). All data are expressed as mean ± SD.

The spared nerve injury (SNI) model was performed as previously described [21]. Briefly, under anesthesia with isoflurane (3.0% induction, 1.5% maintenance), the right sciatic nerve (SN) of mice was uncovered by blunt dissection. The common peroneal (CPN) and tibial nerves (TN) were then tenderly ligated with 5.0 silk and incised together transversely, but the sural nerve was not ligated, and then about 2-mm sections from the distal end of the ligation were removed (Figure 1(a)). For the Sham group, the sciatic nerve was exposed in the same manner, but without any manipulation of the nerves. The mice were returned to their cages after surgery and treated with topical lidocaine (2%) for postoperative pain.

### 2.3. Intrathecal Injection of the Virus

**Figure 2:**
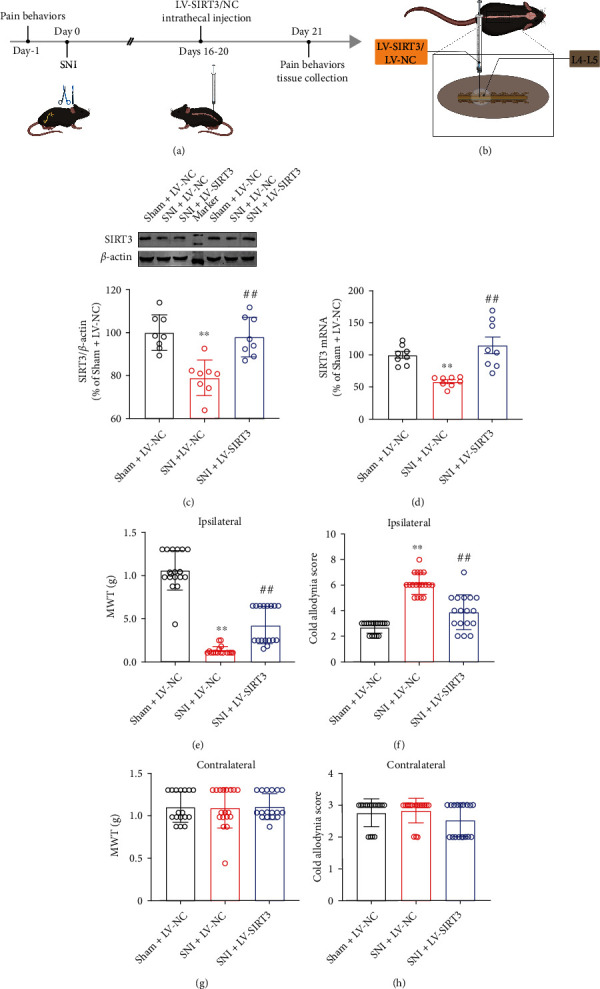
Overexpression of spinal SIRT3 attenuates pain behaviors in the ipsilateral paw of SNI mice. (a) Experimental schedule. (b) Intrathecal injection illustration. (c, d) Protein and mRNA levels of SIRT3 (*n* = 8). (e, f) Mechanical pain and cold pain thresholds in the ipsilateral paw of mice (*n* = 17~18). (g, h) Mechanical pain and cold pain thresholds in the contralateral paw of mice (*n* = 17~18). All data are expressed as mean ± SD. ^∗∗^*P* < 0.01 vs Sham + LV-NC; ^##^*P* < 0.01 vs SNI + LV-NC.

The L4-L6 lumbar spinous process was fixed with one hand, and a Hamilton syringe with a thin needle was inserted at an angle about 20° above the vertebral column into L4 or L5 spinous space with the other hand. The needle was then carefully advanced between the vertebrae as the angle of the syringe was reduced to approximately 10° until an abrupt slight tail flick was observed, which suggested that the needle tip had entered the subarachnoid space (Figure 2(b)). The mouse lentiviral vectors expressing SIRT3 (LV-SIRT3, 4.41 × 10^8^ TU/mL) obtained from OBiO Technology Corp., Ltd. (Shanghai, China) were subsequently injected into the subarachnoid space in a volume of 5 *μ*l for 30 s and the needle is placed in the proper position for 15 s before withdrawal [22, 23]. The lentiviral vectors without SIRT3 (LV-NC) were injected as a control. Both lentiviral vectors were administered intrathecally for 5 consecutive days to the mice (daily from day 16 to day 20 after SNI surgery, 5 *μ*l/d).

### 2.4. Drug Administration

CsA (MCE, New Jersey, USA) working solution in DMSO was diluted by 10% DMSO, 40% PEG300, 5% Tween-80, and 45% normal saline. CsA (20 mg/kg) was injected intraperitoneally into mice 1 d before pain measurement.

### 2.5. Behavioral Tests

The mechanical pain was evaluated by the von Frey test. Briefly, animals were separately placed in clear plastic boxes with a metal mesh floor. After a 30-min acclimatization period, the threshold for paw withdrawal (both ipsilateral and contralateral sides) was measured by calibrated filaments (North Coast Medical, Morgan Hill, California, USA) ranging from 0.008 g to 6 g. The ascending stimulus started at 0.16 g and lasted about 1-2 s each time, with an interval of 5 min between each stimulus. The 50% mechanical withdrawal threshold (MWT) was determined using the up-down method of Chaplan et al. [24].

To assess cold allodynia in the SNI mice, a drop of acetone was applied to the plantar side of the posterior claw. The reaction to acetone was graded on the 4-point scale: [25]:0, no reaction; 1, quick withdrawal, flick, or stamp of the paw; 2, prolonged withdrawal or repeated flicking of the paw; 3, repeated flicking of the paw with licking directed at the paw. The acetone test was alternated three times on each hind paw and the responses were categorically scored. Finally, the sum of the three scores was calculated to evaluate cold pain hypersensitivity in mice. All behavioral tests were administered to an experimenter who was unaware of the treatment group.

### 2.6. Western Blotting Analysis

Western blotting analysis was performed as we described previously [26]. Briefly, the frozen spinal cords were homogenized in lysis buffer (Beyotime Biotech Inc., Jiangsu, China) containing a mixture of protease inhibitors and phenylmethylsulfonyl fluoride. The supernatant was collected after centrifugation at 12000 g under 4°C for 15 min and the protein concentration was determined with a BCA protein assay kit (Thermo Scientific, Massachusetts, USA). Next, the protein samples were separated in equal quantities by SDS-polyacrylamide gel electrophoresis and transferred onto a nitrocellulose membrane (Merck Millipore, Massachusetts, USA). After blocking with 5% skim milk powder, the membrane was incubated with appropriate primary antibodies that against SIRT3 (1 : 1000, D22A3, Cell Signaling Technology, USA), CypD (1 : 1000, ab170190, Abcam, UK), Ac-CypD-K166 (1 : 1000, WG-00545P, ABclonal, China), MnSOD (1 : 1000, ET1701-54, HUABIO, China), or *β*-actin (1 : 1000, AP0060, Bioworld, Louis Park, USA) overnight at 4°C, followed by incubation with the IRDye 800CW second antibody (Li-Cor, Lincoln, Nebraska, USA). Immunoreactive bands were detected using an Infrared Imaging System (Gene Company Limited, Hong Kong, China).

### 2.7. Co-Immunoprecipitation

The interaction between SIRT3 and CypD was analyzed by co-immunoprecipitation (Co-IP) using SIRT3 antibody and CypD antibody. A total of about 500 *μ*g protein extract was incubated with 1 *μ*g of normal IgG (Beyotime Biotech Inc., Jiangsu, China) and 10 *μ*l of fully mixed Protein A/G Agarose (Beyotime Biotech Inc., Jiangsu, China), then shook slowly at 4°C for 2 h to avoid non-specific binding. Next, the supernatant was collected and used for IP after centrifugation at 3000 g for 5 min. Then, 2 *μ*g SIRT3 or CypD primary antibody was added to the supernatant and shaken slowly overnight at 4°C. IgG was used as a negative control. The next day, 20 *μ*l of completely suspended protein A/G Agarose was added to the reaction mixture and left at 4°C for 4 h. Thereafter, the beads containing bound protein were precipitated by centrifugation and washed five times with Co-IP buffer. Subsequently, they were denatured by boiling in SDS-sample buffer. Finally, the obtained immunoprecipitate was subjected to immunoblot analysis. In this process, the membranes were washed with PBST, followed by incubation with the horseradish peroxidase-conjugated secondary antibody for 1 h at room temperature. The bands on the membranes were visualized by chemiluminescence (ECL, Tanon, China) as directed by the manufacturer.

### 2.8. Real-Time Quantitative PCR (RT-qPCR)

RT-qPCR was performed as we described previously [26]. Briefly, total RNA was obtained from spinal cord (L4-L6) tissues with Trizol reagent (Invitrogen, Carlsbad, CA, USA) and transcribed into cDNA using a high-capacity cDNA reverse transcription kit (Applied Biosystems, Foster City, USA). The Roche 480 LightCycler detection system with the GoTaq qPCR Master Mix Kit (Promega, Madison, Wisconsin, USA) was applied to analyze RT-qPCR. The PCR primers used were as follows: SIRT3 5′- AGT GAC ATT GGG CCT GTA GTG -3′ (forward) and 5′- TAC CCT GAA GCC ATC TTT GAA -3′ (reverse); *β*-actin: 5′-AGA GGG AAA TCG TGC GTG AC-3′ (forward) and 5′-CAA TAG TGA TGA CCT GGC CGT-3′ (reverse). The relative mRNA level of SIRT3 was normalized to *β*-actin.

### 2.9. mPTP Opening Assay

Single cell suspensions were prepared from fresh spinal cord tissues, and the fluorescence intensity of Calcein AM was measured using the Mitochondrial Permeability Transition Pore Assay Kit (Beyotime Biotech Inc., Jiangsu, China) according to the manufacturer's protocol. In short, cells and fluorescence quenching solution were incubated in the dark at 37°C for 30 min, and then cells were resuspended with Assay Buffer and the fluorescence intensity of Calcein AM was measured at emission of 517 nm with excitation of 494 nm using a Flow Cytometer (Agilent Technologies, Inc., San Diego, USA). Mean fluorescence intensity (MFI) was used to assess the openness of mPTP and fluorescence intensity is negatively correlated with mPTP opening.

### 2.10. Mitochondrial Membrane Potential (MMP) Level

Mitochondrial membrane potential (*Δψ*m) was measured by Tissue Mitochondria Isolation Kit (Beyotime Biotech Inc., Jiangsu, China) and mitochondrial membrane potential assay kit with JC-1 (Beyotime Biotech Inc., Jiangsu, China). In short, purified mitochondria were extracted from fresh spinal cord tissues with Tissue Mitochondria Isolation Kit. Then, the purified mitochondria and JC-1 staining working solution were incubated at 37°C for 30 min. Finally, the fluorescence intensity of JC-1 monomers (*λ*ex 490 nm, *λ*em 530 nm) and aggregates (*λ*ex 525 nm, *λ*em 590 nm) was tested using a multi-functional microplate reader (Thermo Fisher Scientific Inc., Massachusetts, USA). The results are represented by red (aggregates)/green (monomers) fluorescence ratio.

### 2.11. ROS Production

ROS was measured using a Reactive Oxygen Species Assay Kit (Nanjing Jiancheng Bioengineering Institute, Jiangsu, China) according to the manufacturer's instruction. Briefly, a single cell suspension was prepared from an equal amount of fresh spinal cord tissues, and then cells were incubated with DCFH-DA at 37°C for 30 min. The fluorescence intensity was determined at emission of 525 nm with excitation of 500 nm in a multi-functional microplate reader (Thermo Fisher Scientific Inc., Massachusetts, USA).

### 2.12. MDA Assay

MDA was measured using a Malondialdehyde Kit (Nanjing Jiancheng Bioengineering Institute, Jiangsu, China). Briefly, 10% of the tissue was homogenized on ice, then 200 *μ*l of supernatant was added to reagent 1. Application solutions 2 and 3 were added after vortexing, and both standard and blank tubes were set up and incubated in boiling water for 80 min. The absorbance was measured at 532 nm to calculate the MDA content.

### 2.13. Statistical Analysis

GraphPad Prism v8.0 (GraphPad Software, Inc.) was used for statistical analysis. All the data were presented as mean ± SD. The time course of pain behaviors between groups was analyzed by two-way analysis of variance (ANOVA). For other data, unpaired *t*-test was used for comparison between two groups, and one-way ANOVA with a Bonferroni test was used for multiple comparisons. *P* < 0.05 was considered to be statistically significant.

## 3. Results

### 3.1. Neuropathic Pain Induces Downregulation of SIRT3 in Spinal Cords

The MWT and cold allodynia score were used to evaluate neuropathic pain in mice. As shown in Figures 1(b) and 1(c), we found that the pain thresholds of Sham and SNI model mice were approximately the same at baseline (1 d prior to surgery). Persistent pain-like behaviors occurred only in the ipsilateral (surgical side) paw of the SNI model mice with nerve damage. Moreover, the mechanical allodynia was developed 3 d after SNI surgery and was lowest on d 21. Therefore, we used 21 d postoperatively as the time for pain tests. Interestingly, we found that the ipsilateral paw of mice in the Sham group had reduced MWT on 3 d postoperatively compared to preoperative baseline and returned to normal by day 7. We speculate that this may be due to the inflammatory response caused by the surgery. For cold allodynia, mice in the SNI model exhibited hypersensitivity as early as 3 d postoperatively and persisted at least until day 21. However, no significant pain-like behaviors were observed in the contralateral paw of the Sham group or the SNI model group.

To explore the underlying mechanisms of neuropathic pain, we utilized immunoblotting and RT-qPCR to detect the expression of SIRT3 in the spinal cords of mice. We found that after 21 d of SNI surgery, SIRT3 protein and mRNA levels were significantly downregulated (Figures 1(d) and 1(e)). These results suggested that SIRT3 is involved in the development of neuropathic pain.

### 3.2. SIRT3 Overexpression in Spinal Cords Alleviates SNI-Induced Neuropathic Pain

To confirm the role of SIRT3 in the occurrence of neuropathic pain, LV-SIRT3 was injected into the subarachnoid space of SNI model mice 16 d after SNI surgery, and LV-NC was injected as a control (Figure 2(a)). As shown in Figures 2(c) and 2(d), after injection of LV-SIRT3, the levels of SIRT3 protein and mRNA in spinal cords of SNI model mice were significantly increased, indicating that lentivirus transfection was successful. In addition, compared with the Sham group, we observed that SNI model mice had significantly lower ipsilateral paw MWT and showed a distinct pain response to cold sensation on d 21 after SNI surgery in mice. Mechanical and cold hyperalgesia in the ipsilateral paw were also considerably improved when the SIRT3 of spinal cords was overexpressed in the SNI model mice (Figures 2(e) and 2(f)), with no significant change on the contralateral side (Figures 2(g) and 2(h)). These results suggest that SIRT3 plays a key role in the development of neuropathic pain.

### 3.3. SIRT3 Alleviates Neuropathic Pain by Deacetylating CypD in SNI Mice

**Figure 3:**
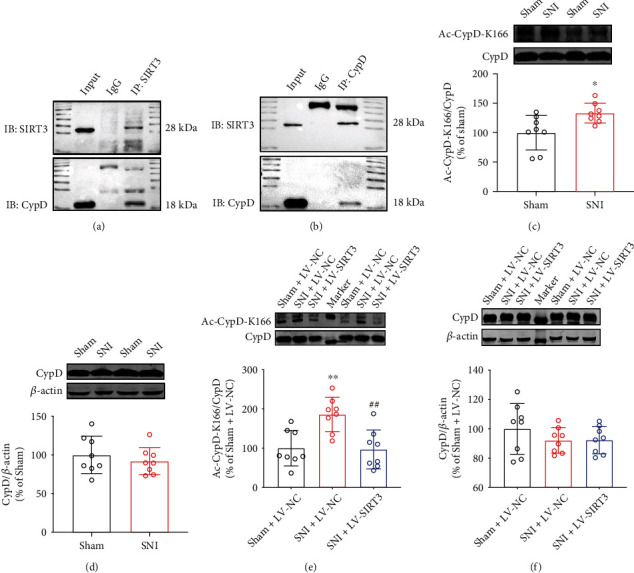
SIRT3 mediates the deacetylation of CypD in SNI mice. (a) Co-IP for SIRT3. (b) Co-IP for CypD. (c, d) Changes of Ac-CypD-K166 and CypD expressions in the spinal cords of SNI mice (^∗^*P* < 0.05 vs Sham). (e, f) Effect of SIRT3 overexpression on the levels of Ac-CypD-K166 and CypD in the spinal cords of SNI mice (^∗∗^*P* < 0.01 vs Sham + LV-NC; ^##^*P* < 0.01 vs SNI + LV-NC). All data are expressed as mean ± SD. *n* =8.

It has been demonstrated that SIRT3 can deacetylate CypD, an important mitochondrial matrix protein, improving cognitive dysfunction, myocardial reperfusion injury, and cardiac hypertrophy [19, 27, 28]. Especially, lysine 166 (K166) on CypD has been shown to be a deacetylating target of SIRT3 [19]. Therefore, to investigate the underlying mechanisms for SIRT3 to alleviate neuropathic pain, firstly, we detected the relationship between SIRT3 and CypD. The anti-SIRT3 and anti-CypD antibodies were used for Co-IP analysis. A strong positive CypD signal was observed in SIRT3 immunoprecipitation (Figure 3(a)), and similarly, a positive signal of SIRT3 was observed in CypD immunoprecipitation (Figure 3(b)), indicating that SIRT3 interacts with CypD. Secondly, we found that after 21 d of SNI surgery, Ac-CypD-K166 protein expression was clearly upregulated in the SNI model mice compared with the Sham group (Figure 3(c)). However, the total CypD protein level was unchanged (Figure 3(d)). When we injected LV-SIRT3, the expression of Ac-CypD-K166 decreased in the spinal cords of SNI model mice (Figure 3(e)), but there was no significant change in total CypD protein (Figure 3(f)). These results suggest that SIRT3 can mediate the deacetylation of CypD at lysine 166, which may be involved in the development of neuropathic pain.

### 3.4. Point Mutation of K166 to Arginine on CypD (CypD-K166R) Abrogates SNI-Induced Neuropathic Pain

**Figure 4:**
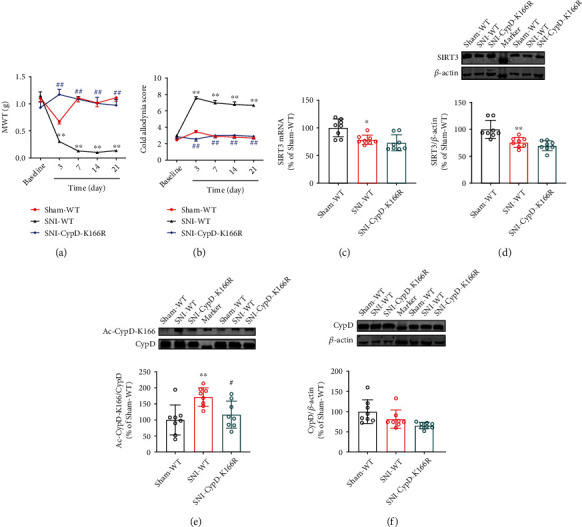
Point mutation of K166 to arginine on CypD (CypD-K166R) abrogates SNI-induced neuropathic pain. (a, b) Mechanical pain and cold pain thresholds in CypD-K166R point mutant mice before and 3, 7, 14, and 21 d after SNI surgery (*n* =9. ^∗∗^*P* < 0.01 vs Sham-WT; ^##^*P* < 0.01 vs SNI-WT). (c–f) Changes in the levels of SIRT3, Ac-CypD-K166, and CypD in CypD-K166R point mutant mice after SNI surgery (*n* =8. ^∗^*P* < 0.05, ^∗∗^*P* < 0.01 vs Sham-WT; ^#^*P* < 0.05 vs SNI-WT). All data are expressed as mean ± SD.

To further confirm the role of acetylation of CypD-K166 in the development of neuropathic pain, we used CypD-K166R point mutant mice to construct a SNI model and to observe the change of pain behaviors after specific mutation of the key site of CypD acetylation. Meanwhile, a SNI model was constructed from wild-type mice using as a control. As shown in Figures 4(a) and 4(b), wild-type mice in the SNI model group exhibited strong pain allodynia in the ipsilateral paw postoperatively compared with wild-type mice in the Sham group. CypD-K166R point mutant mice had significantly higher mechanical and cold pain thresholds in the ipsilateral paw compared to wild-type mice in the SNI model group. Compared with the preoperative baseline, CypD-K166R point mutant mice showed no visible changes in pain thresholds. Moreover, compared with wild-type mice in the SNI model group, CypD-K166R point mutant mice showed no significant changes in CypD and SIRT3 levels after SNI surgery, and Ac-CypD-K166 expression was not upregulated (Figures 4(c)–4(f)). These results suggest that acetylation of CypD at lysine 166 is a critical link in the development of neuropathic pain.

### 3.5. SIRT3 Overexpression Inhibits Ac-CypD-Mediated mPTP Opening and Mitochondrial Dysfunction

**Figure 5:**
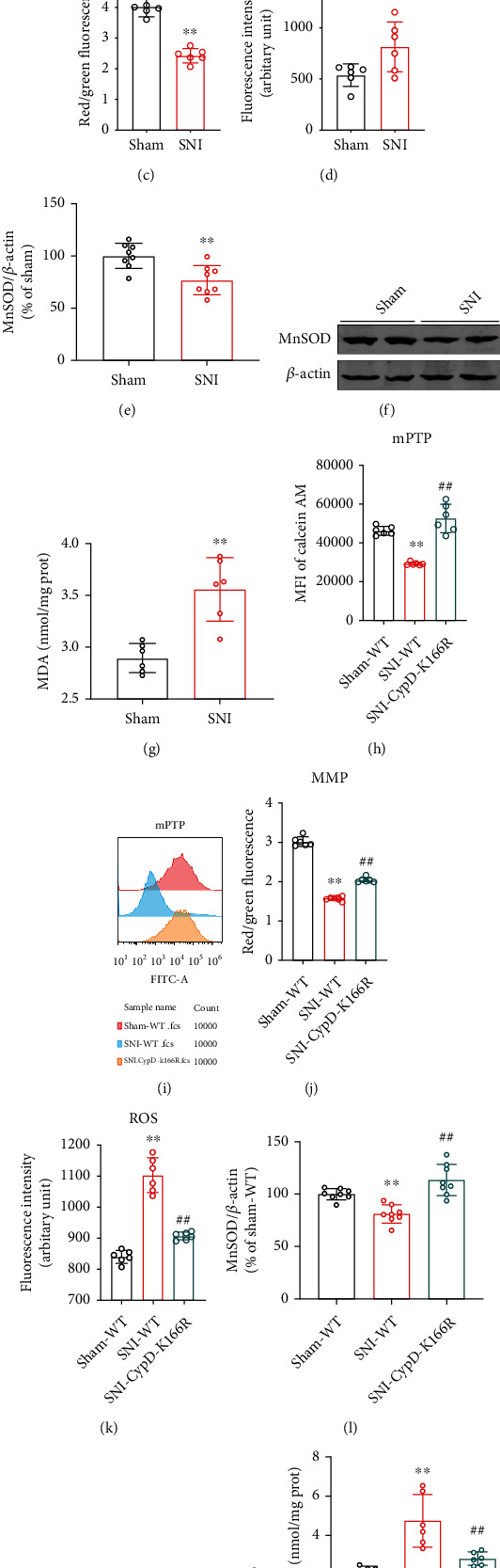
CypD-K166 acetylation is necessary for SNI-induced mitochondrial dysfunction and oxidative stress. (a, b) Open levels of mitochondrial permeability transition pore (mPTP) were evaluated by Flow Cytometry. Mean fluorescence intensity (MFI) is negatively correlated with mPTP opening (*n* = 6. ^∗∗^*P* < 0.01 vs Sham). (c) Mitochondrial Membrane Potential (MMP) in SNI model mice (*n* = 6. ^∗∗^*P* < 0.01 vs Sham). (d) Reactive oxygen species (ROS) levels in SNI model mice (*n* = 6. ^∗^*P* < 0.05 vs Sham). (e, f) Western blotting analysis for MnSOD levels (*n* =8. ^∗∗^*P* < 0.01 vs Sham). (g) MDA levels in SNI model mice (*n* = 6. ^∗∗^*P* < 0.01 vs Sham). (h–n) Changes in mPTP, MMP, ROS, MnSOD, and MDA levels in CypD-K166R point mutant mice after SNI surgery (*n* = 6-8. ^∗∗^*P* < 0.01 vs Sham-WT; ^##^*P* < 0.01 vs SNI-WT). All data are expressed as mean ± SD.

**Figure 6:**
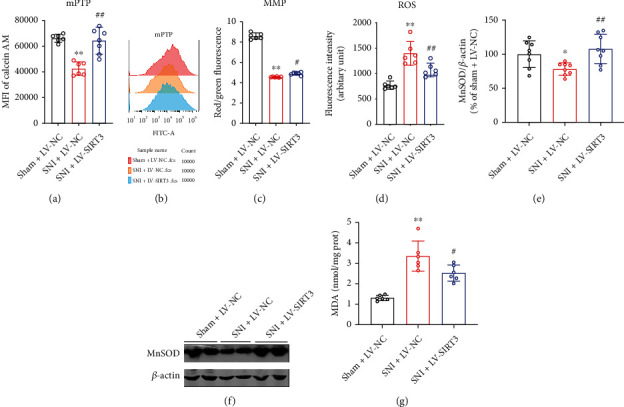
SIRT3 overexpression improves SNI-induced mitochondrial dysfunction and oxidative stress. (a, b) Open levels of mitochondrial permeability transition pore (mPTP) were evaluated by Flow Cytometry. Mean fluorescence intensity (MFI) is negatively correlated with mPTP opening (*n* = 6. ^∗∗^*P* < 0.01 vs Sham). (c) Mitochondrial Membrane Potential (MMP) (*n* = 6. ^∗∗^*P* < 0.01 vs Sham). (d) Reactive oxygen species (ROS) levels (*n* = 6. ^∗^*P* < 0.05 vs Sham). (e, f) Western blotting analysis for MnSOD levels (*n* =8. ^∗∗^*P* < 0.01 vs Sham). (g) MDA levels (*n* = 6. ^∗∗^*P* < 0.01 vs Sham). All data are expressed as mean ± SD.

CypD has been reported to be an important component of the mPTP, and the upregulation of Ac-CypD can cause ANT conformational changes, leading to an increase in mPTP opening, which in turn causes mitochondrial dysfunction and oxidative stress [13, 29–31]. Therefore, to investigate the mechanism of the acetylation of CypD-K166 in SIRT3-mediated neuropathic pain, we measured mPTP, MMP, ROS, MDA, and MnSOD levels in the spinal cords of mice. We found that mPTP opening was increased, MMP was decreased, ROS and MDA production was significantly increased, and the protein expression level of MnSOD was markedly downregulated in mice of the SNI model group compared to the Sham group (Figures 5(a)–5(g)). However, CypD-K166R point mutant mice did not exhibit similar results after SNI surgery (Figures 5(h)–5(n)), suggesting that the acetylation of CypD-K166 is necessary for SNI-induced mitochondrial dysfunction and oxidative stress. In addition, we also found that SIRT3 overexpression could reverse SNI-induced increase of mPTP opening, ROS and MDA levels, and decrease of MMP and MnSOD (Figures 6(a)–6(g)). These results suggest that the overexpression of SIRT3 in the spinal cords of SNI model mice could improve mitochondrial dysfunction and inhibit oxidative stress by inhibiting Ac-CypD-mediated mPTP opening.

### 3.6. Inhibiting mPTP Opening Improves Mitochondrial Function and Neuropathic Pain

**Figure 7:**
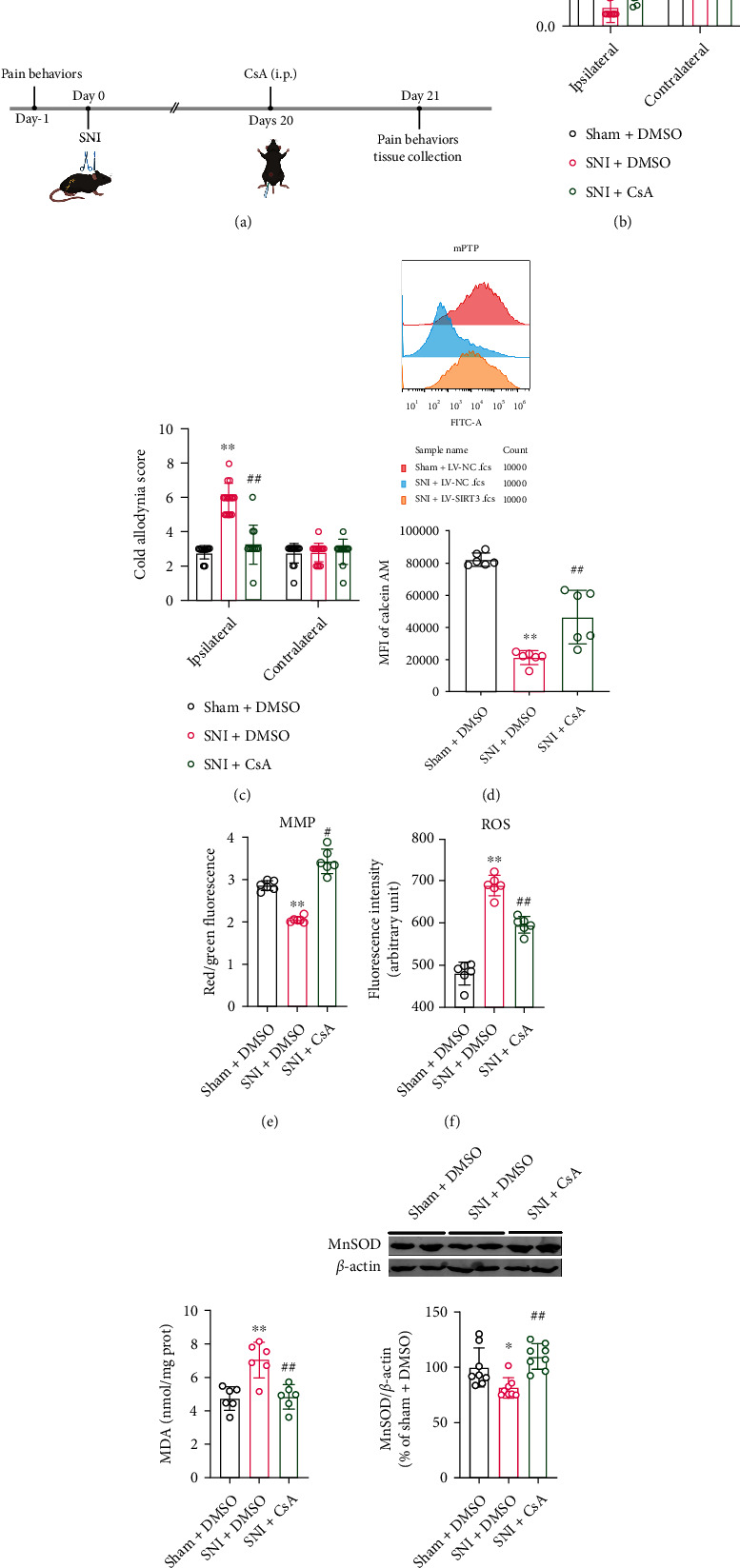
Inhibiting mPTP opening improves mitochondrial function and neuropathic pain. (a) Experimental schedule. (b, c) Mechanical pain and cold pain thresholds in SNI model mice after CsA injection (*n* = 12-18. ^∗∗^*P* < 0.01 vs Sham + DMSO; ^##^*P* < 0.01 vs SNI + DMSO). (d–h) mPTP opening, MMP, ROS, MDA, and MnSOD levels in SNI model mice after injection of CsA (*n* = 6-8. ^∗∗^*P* < 0.01 vs Sham + DMSO; ^##^*P* < 0.01 vs SNI + DMSO). All data are expressed as mean ± SD.

To further verify the role of mPTP in the regulation of neuropathic pain, the mPTP blocker CsA (20 mg/kg) was injected intraperitoneally on day 20 after SNI surgery in mice (Figure 7(a)), and behavioral tests were performed on day 21 after surgery. The results showed that CsA significantly alleviated mechanical and cold hyperalgesia of ipsilateral paw in SNI model mice, with no effect on that of the contralateral side (Figures 7(b) and 7(c)). Meanwhile, CsA significantly decreased mPTP opening, increased MMP and MnSOD, and downregulated ROS and MDA levels in SNI model mice (Figures 7(d)–7(h)). These results indicate that inhibiting mPTP opening improves mitochondrial function and neuropathic pain in SNI mice.

## 4. Discussion

**Figure 8:**
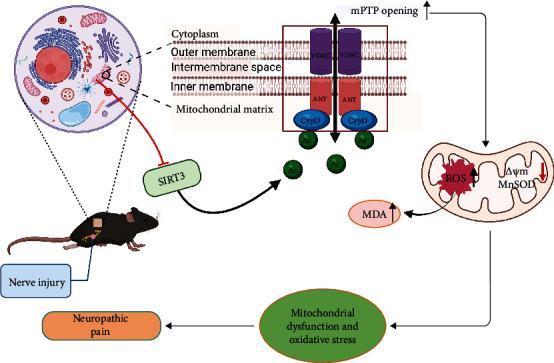
The diagram illustrates the role of SIRT3 in the development of neuropathic pain. After nerve injury, the loss of SIRT3 function leads to CypD acetylation. As a result, mitochondrial permeability transition pore (mPTP) opening increases, mitochondrial membrane potential (MMP) decreases, and reactive oxygen species (ROS) accumulate, contributing to mitochondrial dysfunction and oxidative stress, ultimately leading to neuropathic pain.

Our study showed that SIRT3 expression was obviously downregulated and Ac-CypD-K166 was significantly upregulated in the spinal cords of SNI model mice. Spinal cord SIRT3 overexpression attenuated acetylation of CypD at lysine 166 and reversed pain hypersensitivity in ipsilateral paw of SNI model mice. Further studies showed that CypD deacetylation ameliorated mitochondrial dysfunction and inhibited oxidative stress, thereby reducing allodynia and hyperalgesia in the SNI model mice. In addition, intraperitoneal injection of CsA, a specific inhibitor of mPTP, protected mitochondrial function and improved pain-like behaviors in mice. In conclusion, this study demonstrates that SIRT3 is a key epigenetic regulatory protein in the development of neuropathic pain. SIRT3-mediated CypD deacetylation can ameliorate mitochondrial dysfunction and inhibit oxidative stress by inhibiting mPTP opening, further reducing neuropathic pain (Figure 8).

Neuropathic pain is a complex and heterogeneous group of disease states with few clear answers in terms of treatment options at present [32]. The mechanisms by which neuropathic pain occurs are still unclear, and a growing number of studies suggest that mitochondrial dysfunction and oxidative stress are involved in the development and maintenance of neuropathic pain [5, 6]. For example, mitoxantrone reduces neuropathic pain by improving mitochondrial dysfunction and inhibiting oxidative stress and apoptosis [5]. SIRT3 is mainly located in mitochondria [33]. Studies have shown that SIRT3 is closely associated with the regulation of oxidative stress-related mitochondrial damage in cardiac ischemia-reperfusion injury and Alzheimer's disease [19, 34]. In a previous study [26], we have shown that SIRT3 can upregulate antioxidant genes such as superoxide dismutase (SOD). In addition, ROS levels were significantly upregulated in germ cell lines of mice with SIRT3 knocked out, while ROS production was inhibited in cell lines that SIRT3 was overexpressed [35]. In mice with hyperoxia-induced acute lung injury model, overexpression of SIRT3 increased the expression of manganese-containing superoxide dismutase (MnSOD), a kind of mitochondrial antioxidant enzyme, enhancing its activity and inhibiting oxidative stress [36]. These results demonstrate that SIRT3 can regulate oxidative stress in the organism. Some other studies have shown that SIRT3 can also activate some key enzymes through its deacetylation activity, thus enhancing mitochondrial oxidative respiration and ensuring the stability of mitochondrial energy metabolism [37, 38]. Therefore, we speculate that SIRT3 may be involved in the development of neuropathic pain by improving mitochondrial dysfunction and inhibiting oxidative stress. To confirm this hypothesis, we measured the levels of mPTP, MMP, ROS, MnSOD, and MDA in the present study. The results showed that both protein and mRNA levels of SIRT3 were decreased in the spinal cords of mice in the SNI model group, and the mPTP opening and ROS levels were increased, while MMP was decreased. In addition, we also found that MnSOD protein levels were significantly downregulated and MDA levels were upregulated in the spinal cords of SNI mice. These changes were reversed when we overexpressed spinal SIRT3 by intrathecal injection of LV-SIRT3. In addition, SIRT3 overexpression improved pain allodynia in SNI model mice. These results suggest that SIRT3 can mediate the development of neuropathic pain at the spinal cord level by ameliorating mitochondrial dysfunction and inhibiting oxidative stress.

Epigenetic modifications including DNA methylation, histone modifications, and RNA modifications have been reported to play key roles in the development and maintenance of neuropathic pain [39–41]. As an important deacetylase, SIRT3 has been shown to regulate mitochondrial oxidative stress by deacetylating mitochondria-related proteins [17, 42]. Moreover, an imbalance between mitochondrial acetylation and decreased SIRT3 activity can lead to mitochondrial hyperacetylation, inducing mitochondrial dysfunction and oxidative stress [43]. Especially, a large body of evidence demonstrates that SIRT3 can deacetylate CypD, an important mitochondrial matrix protein, improving cognitive dysfunction and myocardial reperfusion injury [27, 28]. Therefore, to explore the potential downstream mechanisms for SIRT3 to attenuate neuropathic pain, we observed the role of SIRT3 in CypD deacetylation. Our results showed that the acetylation level of CypD was significantly elevated in the SNI model mice, and overexpression of SIRT3 obviously reduced the acetylation level of CypD. In addition, we further confirmed the interaction between SIRT3 and CypD by Co-IP. The above results suggest that SIRT3 is involved in neuropathic pain by deacetylating CypD. CypD is known to induce mPTP opening and subsequently induce mitochondrial dysfunction by altering the conformation of ANT through acetylation [13, 44–46]. Furthermore, T. Bochaton et al. [27] found that SIRT3 prevents CypD from binding to ANT in HeLa cells and that destruction of this interplay depends on the deacetylation of CypD by SIRT3. This reinforces this point. Therefore, we suggest that spinal SIRT3-mediated CypD deacetylation may inhibit mPTP opening, improve mitochondrial oxidative stress, and ultimately attenuate pain hypersensitivity in SNI mice. To test this idea, we constructed SNI models on CypD-K166R mutant mice. Consistent with our predictions, wild-type mice in the SNI model group showed upregulated Ac-CypD-K166 protein expression, increased mPTP opening, loss of membrane potential, and excessive accumulation of ROS and MDA accompanied by significant downregulation of MnSOD. However, these changes did not occur on CypD-K166R mutant mice. This may result from a dramatic decrease in the acetylation level of CypD after mutation at position 166. There is evidence [27] that hypoxia-reoxygenation of cardiomyocytes also increases CypD acetylation, and its deacetylation decreases the likelihood of mPTP opening, and interestingly, the CypD acetylation mimic CypD-KQ decreases calcium retention and increases cell death in mouse embryonic fibroblasts, whereas the non-acetylated CypD mutant CypD-KR has the opposite effect, which is consistent with our results. In addition, mice did not exhibit pain allodynia and hyperalgesia after mutation of CypD-K166. These results suggest that acetylation of CypD at lysine 166 is an important link in the development of neuropathic pain.

mPTP is a non-specific pore [11], and mPTP opening can induce increased mitochondrial membrane permeability, mitochondrial swelling, decreased membrane potential, and the accumulation of more ROS [47, 48]. It has been suggested that mPTP is required for the release of mitochondrial ROS [49]. The mPTP is regulated by redox, and the formation of the VDAC-ANT-CypD complex under oxidative stress triggers the opening of mPTP and promotes the release of ROS into the cytoplasm [50–53]. Furthermore, mPTP plays an important role in a variety of neurodegenerative diseases [54], but little is known about the role of mPTP in the development of neuropathic pain. However, in neuropathic pain rats, the ratio of cytochrome c- (Cytc-) positive neurons and the level of Cytc have been found to increase significantly, while Cytc is released from mitochondria by mPTP opening [7, 55]. Moreover, CsA, a well-known specific inhibitor of mPTP, can decrease the release of Cytc and increase mechanical threshold [7]. Therefore, we speculated that mPTP opening is likely to be closely related to the development of neuropathic pain. In our study, we found that after injection of CsA, mPTP opening was significantly reduced, mitochondrial function was obviously improved, and ROS production was clearly reduced. More importantly, CsA attenuated the pain allodynia and hyperalgesia in SNI model mice. These results demonstrate that mPTP is an important regulator in neuropathic pain.

## 5. Conclusion

In conclusion, our current study shows that neuropathic pain in mice disrupts the function of spinal SIRT3, leading to increased acetylation of CypD, which leads to increased mPTP opening and further induction of mitochondrial dysfunction and oxidative stress. These results suggest that SIRT3-mediated CypD deacetylation can serve as a potential drug target for the treatment of neuropathic pain.

## Data Availability

The data used to support the findings of this study are available from the corresponding author upon reasonable request.
